# Anti-leucine rich glioma inactivated 1 protein and anti-N-methyl-D-aspartate receptor encephalitis show distinct patterns of brain glucose metabolism in ^18^F-fluoro-2-deoxy-d-glucose positron emission tomography

**DOI:** 10.1186/1471-2377-14-136

**Published:** 2014-06-20

**Authors:** Florian Wegner, Florian Wilke, Peter Raab, Said Ben Tayeb, Anna-Lena Boeck, Cathleen Haense, Corinna Trebst, Elke Voss, Christoph Schrader, Frank Logemann, Jörg Ahrens, Andreas Leffler, Rea Rodriguez-Raecke, Reinhard Dengler, Lilli Geworski, Frank M Bengel, Georg Berding, Martin Stangel, Elham Nabavi

**Affiliations:** 1 Department of Neurology, Hannover Medical School, Carl-Neuberg-Str. 1, 30625 Hannover, Germany; 2 Department of Medical Physics, Hannover Medical School, Carl-Neuberg-Str. 1, 30625 Hannover, Germany; 3 Institute of Neuroradiology, Hannover Medical School, Carl-Neuberg-Str. 1, 30625 Hannover, Germany; 4 Department of Nuclear Medicine, Hannover Medical School, Carl-Neuberg-Str. 1, 30625 Hannover, Germany; 5 Department of Anaesthesia and Critical Care Medicine, Hannover Medical School, Carl-Neuberg-Str. 1, 30625 Hannover, Germany

**Keywords:** Anti- leucine rich glioma inactivated 1 protein (LGI1), Anti- N-methyl-D-aspartate (NMDA) receptor antibody, Paraneoplastic syndrome, Autoimmune limbic encephalitis, Brain glucose metabolism, ^18^F-fluoro-2-deoxy-d-glucose positron emission tomography (FDG-PET)

## Abstract

**Background:**

Pathogenic autoantibodies targeting the recently identified leucine rich glioma inactivated 1 protein and the subunit 1 of the N-methyl-D-aspartate receptor induce autoimmune encephalitis. A comparison of brain metabolic patterns in ^18^F-fluoro-2-deoxy-d-glucose positron emission tomography of anti-leucine rich glioma inactivated 1 protein and anti-N-methyl-D-aspartate receptor encephalitis patients has not been performed yet and shall be helpful in differentiating these two most common forms of autoimmune encephalitis.

**Methods:**

The brain ^18^F-fluoro-2-deoxy-d-glucose uptake from whole-body positron emission tomography of six anti-N-methyl-D-aspartate receptor encephalitis patients and four patients with anti-leucine rich glioma inactivated 1 protein encephalitis admitted to Hannover Medical School between 2008 and 2012 was retrospectively analyzed and compared to matched controls.

**Results:**

Group analysis of anti-N-methyl-D-aspartate encephalitis patients demonstrated regionally limited hypermetabolism in frontotemporal areas contrasting an extensive hypometabolism in parietal lobes, whereas the anti-leucine rich glioma inactivated 1 protein syndrome was characterized by hypermetabolism in cerebellar, basal ganglia, occipital and precentral areas and minor frontomesial hypometabolism.

**Conclusions:**

This retrospective ^18^F-fluoro-2-deoxy-d-glucose positron emission tomography study provides novel evidence for distinct brain metabolic patterns in patients with anti-leucine rich glioma inactivated 1 protein and anti-N-methyl-D-aspartate receptor encephalitis.

## Background

Limbic encephalitis involving the temporomedial lobes and amygdalae is characterized by subacute memory impairment, seizures and neuropsychiatric symptoms with variable evidence of cerebrospinal fluid inflammation, anti-neuronal antibodies and a paraneoplastic origin [1-4].

The two most common targets of encephalitis associated pathogenic autoantibodies are the recently identified leucine rich glioma inactivated 1 protein (LGI1), which is extracellularly complexed with voltage-gated potassium channels (VGKC), and the subunit 1 (NR1) of the N-methyl-D-aspartate (NMDA) receptor [5-8]. LGI1 antibodies associated with limbic encephalits specifically inhibited the ligand-receptor interaction between LGI1 and ADAM22 (disintegrin and metalloproteinase domain-containing protein 22) and reversibly reduced synaptic AMPA (α-amino-3-hydroxy-5-methyl-4-isoxazolepropionic acid) receptor clusters in rat hippocampal neurons [9]. Cerebrospinal fluid samples or purified immunoglobulin G (IgG) from patients with anti-NMDA receptor encephalitis led to a marked reduction of NR1 (and NR2B) surface expression and NMDA receptor mediated currents in hippocampal cultures [10,11] as well as to increased corticomotor hyperexcitability in rats [12,13].

While the anti-LGI1 syndrome predominantly reminds of a classic limbic encephalitis with amnesia, (faciobrachial dystonic) seizures and psychiatric manifestations, the anti-NMDA receptor encephalitis is characterized by memory deficits, psychiatric symptoms with psychotic and catatonic features, language disintegration, dyskinetic movements, seizures, decreased consciousness, autonomic and breathing instability that often requires intensive care treatment [6,7,13-15]. However, there may be clinical overlaps of the two entities. A tumor association is infrequently found in the anti-LGI1 syndrome, whereas ovarian or other teratoma were diagnosed in up to 50% of anti-NMDA receptor encephalitis patients [4,6,13,16].

In order to detect underlying tumor manifestations, whole-body ^18^F-fluoro-2-deoxy-d-glucose positron emission tomography (FDG-PET) is performed in many patients with limbic encephalitis, whereby cerebral FDG uptake can also be determined. Some case reports have published FDG-PET data showing brain metabolic abnormalities of varying degree and localization in adult patients with anti-LGI1 [17] or anti-NMDA receptor encephalitis [16,18-22]. Recently, an FDG-PET study revealed a frontal and temporal hypermetabolism associated with occipital hypometabolism in six patients with anti-NMDA receptor encephalitis [23], while a hypermetabolism in the medial temporal lobes and basal ganglia was detected in ten anti-LGI1 encephalitis patients [24]. The aim of our study was to compare cerebral FDG uptake of whole-body FDG-PET imaging in patients with anti-LGI1 and anti-NMDA receptor encephalitis for detailed analysis of brain metabolic disease patterns that may lead to an improved diagnostic accuracy.

## Methods

### Patients and controls

We obtained an approval from the local Ethics Committee of Hannover Medical School (No. 1625–2012) and patients or their carers gave their written informed consent.

The brain FDG uptake from whole-body FDG-PETs of six anti-NMDA receptor encephalitis patients (6 females, median age 36.5, interquartile range 30.5-47.25, Table 1) and four anti-LGI1 encephalitis patients (4 males; median age 68.0, interquartile range 61–72.75, Table 2) admitted to Hannover Medical School between 2008 and 2012 was retrospectively analyzed.

**Table 1:** Clinical, diagnostic and treatment data of patients with anti-N-methyl-D-aspartate receptor encephalitis

**Patient number**	**1**	**2**	**3**	**4**	**5**	**6**
Psychiatric symptoms	++	++	+	+	+	++
Generalized Seizures	++	++	++	+	+	+
Serum anti-NMDA receptor IgG titer	1:320	1:1000	-	1:1000	1:800	-
CSF anti-NMDA receptor IgG titer	1:10	1:100	1:10	-	1:100	1:100
CSF leukocytes/μl	154	2	27	2	270	19
CSF oligoclonal bands	+	+	-	+	+	+
MRI lesions	Hippocampal T2-hyper-intensities + bilateral diffusion elevation	-	unspecific	Subcortical T2-hyper-intensities + contrast enhance-ment	-	-
Clinical onset to PET and treatment in months	1	4	1	15	4	7
mRS at time of PET	5	5	5	4	4	3
PET in propofol narcosis	+	+	+	+	+	-
Intensive care treatment	+	+	+	-	+	-
Methylprednisolone	+	+	+	+	+	+
Plasmapheresis	+	+	-	+	+	-
Immunoglobulins	+	+	+	-	-	-
Immunosuppressive therapy	Rituxim. Cycloph.	Cycloph.	Rituxim. Cycloph.	Azathiopr.	Rituxim. Cycloph.	-
Oophorectomy	+	+	+	+	+	-
Ovarian teratoma	+	+	-	-	-	-
mRS at follow up (months after PET)	4	1	1	4	1	0
	(40)	(40)	(28)	(8)	(16)	(36)

*Abbreviations:* Azathioprine (Azathiopr.), Cyclophosphamide (Cycloph.), cerebrospinal fluid (CSF), computed tomography (CT), positron emission tomography (PET), immunoglobulin G (IgG), magnet resonance imaging (MRI), modified Rankin Scale (mRS), N-methyl-D-aspartate (NMDA), Rituximab (Rituxim.).

**Table 2:** Clinical, diagnostic and treatment data of patients with anti-leucine rich glioma inactivated 1 protein encephalitis

**Patient number**	**1**	**2**	**3**	**4**
Psychiatric symptoms	-	+	-	-
Cognitive deficits	+ (working memory, attention, construction)	++	+	+
		(memory)	(memory)	(memory)
Focal Seizures	+ (dystonic faciobrachial)	+	+	+ (dystonic faciobrachial)
Anti-LGI1 IgG titer in serum	1:100	1:100	1:100	1:100
Anti-LGI1 IgG titer in CSF	-	-	-	-
CSF leukocytes/μl	2	3	2	2
CSF oligoclonal bands	-	-	-	-
MRI lesions	unspecific	bitemporal T2-hyperintensities	-	-
Clinical onset to PET and treatment in months	6	1	12	2
mRS at time of PET	2	2	2	2
PET in narcosis	-	-	-	-
Evidence of Tumor	-	-	-	-
Intensive care treatment	-	-	-	-
Methylprednisolone	+	+	+	+
Plasmapheresis	+ (3 months interval)	+	+	-
Immunoglobulins	-	-	+	+
Immunosuppressive therapy	-	-	Cyclophospha-mide	-
mRS at follow up (months after PET)	1	0	3	0
	(31)	(24)	(9)	(18)

*Abbreviations:* Cerebrospinal fluid (CSF), positron emission tomography (PET), immunoglobulin G (IgG), leucine rich glioma inactivated 1 protein (LGI1), magnet resonance imaging (MRI), modified Rankin Scale (mRS).

Diagnostic criteria were the above mentioned typical clinical symptoms of limbic encephalitis and detection of either anti-LGI1 or anti-NMDA receptor IgG titer in serum or cerebrospinal fluid (CSF) of at least 1:10. Antibody detection was performed using Mosaik Biochips (Euroimmun, Lübeck, Germany) as described previously [25]. The clinical severity at the time of PET investigation and follow up was staged using the modified Rankin Scale (mRS). All patients with anti-LGI1 syndrome but only one patient with anti-NMDA receptor encephalitis were able to lie quietly in the PET scanner, therefore, five of the latter patients had to be investigated in propofol narcosis. The patient imaging data were compared to separate age and sex matched control groups without neuropsychiatric diseases who received PET due to an extracerebral neoplasm (anti-NMDA controls: 5 females, median age 40.0, interquartile range 37.5-46, anti-LGI1 controls: 4 males/2 females, median age 61.0, interquartile range 58.75-64.5). All controls were investigated without narcosis using the same PET acquisition protocol.

### FDG-PET

For positron emission tomography a Biograph LSO Duo PET/CT (Siemens, Erlangen, Germany) was used. One hour before scanning 350 MBq ^18^F-fluoro-2-deoxy-d-glucose (FDG) were injected intravenously. In patients not able to avoid movements during the scan while awake, anesthesia was started shortly before imaging, i.e. after a sufficient uptake phase of FDG. First a whole body low-dose computed tomography (CT)-scan was acquired from the mid-tight to the head using adjusted tube current (Care Dose4D). Subsequently emission scanning was performed in 3D mode typically over 8 bed positions of 4 minutes each. For the present study brain images were reconstructed separately with a matrix size of 256 × 256 and zoom factor 2 using a 2D OSEM (ordered subset expectation maximization) algorithm with 4 iterations and 8 subsets, smoothing with a 5-mm FWHM (full width at half maximum) filter and applying CT based attenuation correction.

### Statistical parametric mapping and volume of interest analysis

Brain images were spatially normalized into the stereotatic standard space according to Montreal Neurological Institute using the Statistical parametric mapping 2 (SPM2) FDG brain template with default parameter settings (statistical parametric mapping, Wellcome Trust Centre for Neuroimaging, London, UK). Thereafter each data set was smoothed with an isotropic 3D Gaussian filter kernel with 10-mm FWHM. In group comparisons (e.g. patients vs. controls) proportional scaling to the cerebral global mean was employed and the two sample t-test to detect voxels/regions with either decreased or increased FDG uptake i.e. glucose metabolism. In single-subject analyses effects were assessed for each patient individually. Moreover, antibody titers and clinical scores were correlated to glucose metabolism as covariates at a group level. Statistical inferences were based on a p-value of 0.005 uncorrected for multiple comparisons and an extent threshold of 30 voxels. We additionally performed group analyses using pons and motor cortex as reference region for scaling leading to essentially the same results. For spatial assignment of significant changes in SPM analyses volume of interest (VOI) templates according to Cyceron or reflecting Brodmann areas were used. In order to detect changes in larger brain regions, small VOIs according to Cyceron were summarized to large VOIs (Additional file 1: Table S1) and statistical analyses for group differences (t-test) and correlations (linear regression analysis) were performed using JMP10 software (SAS Institute). A p-value < 0.05 was used for statistical inferences.

## Results

### Patient and magnet resonance imaging properties

**Figure 1:**
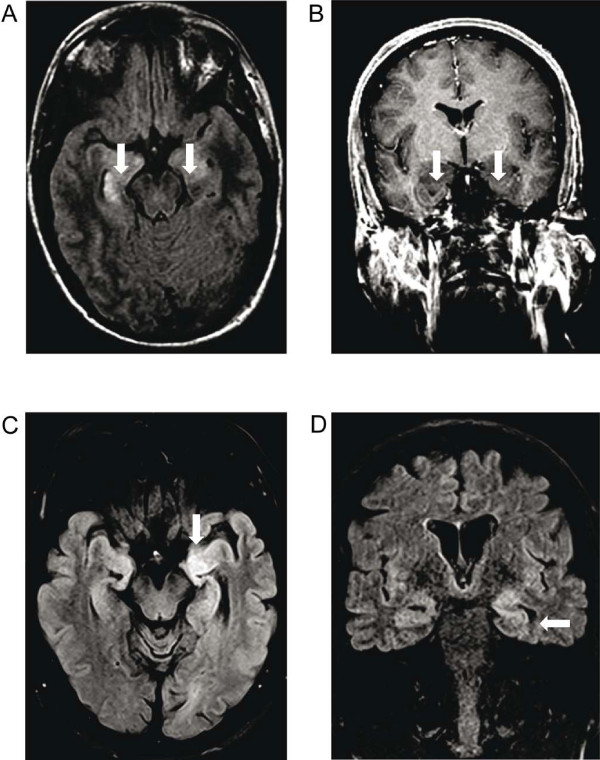
**Magnet resonance imaging in anti-N-methyl-D-aspartate receptor and anti-leucine rich glioma inactivated 1 protein encephalitis patients.** Magnet resonance images from patient 1 with anti-N-methyl-D-aspartate receptor encephalitis **(A-B)** and patient 2 with anti-leucine rich glioma inactivated 1 protein syndrome **(C-D)** are shown. In the top row (A - axial T2w-fluid attenuated inversion recovery image, B - coronal T1 weighted image with contrast enhancement) bilateral T2w-fluid attenuated inversion recovery signal hyperintensities of the corpus amygdaloideum and of the right hippocampus (arrows) are demonstrated. There is no contrast enhancement in the area of the T1w-signal drop of the medial temporal lobe bilaterally (b, arrows). In the bottom row (C – axial and d – coronal T2w-fluid attenuated inversion recovery image) there is a marked T2w-fluid attenuated inversion recovery hyperintensity of the left medial temporal lobe (C – arrow), which also extends to the hippocampus (D – arrow).

The demographic and clinical data of patients as well as their diagnostic procedures and therapy are summarized in Tables 1 and 2. The variability of PET time points in anti-NMDA encephalitis patients was caused mainly by late admission to Hannover Medical School in the disease course of some patients, however, 5/6 patients were considered to be at the clinical peak of encephalitis at the time of PET. They presented with typical signs and symptoms including generalized seizures which were treated successfully with antiepileptic drugs before PET investigation. At the time of PET the electroencephalography (EEG) did not record epilepsy specific potentials and the modified Rankin Scale (mRS) varied between 3 and 5 (median 4.5, interquartile range 3.75-5.0, n = 6).PET metabolic abnormalities of anti-NMDA receptor encephalitis patients did not correspond to magnet resonance imaging (MRI) lesions that were shown in 4/6 patients: Hippocampal T2-hyperintensities bilaterally with diffusion elevation (patient 1, Figure 1A-B), small single unspecific left paraventricular T2-hyperintensity without contrast enhancement or diffusion restriction (patient 3), multiple very small subcortical T2-hyperintensities with T1-contrast enhancement (patient 4) and faint T2-hyperintensities in both cingular gyri without contrast enhancement (patient 5). In patients 2 and 6 MRI did not display any lesions.

Although ovarian PET and ultrasound were unsuspicious, an exploratory oophorectomy [6,26,27] was initiated in 5/6 patients (mRS ≥ 4) after the consent of the caring person had been obtained. Histopathologically a microscopic mature ovarian teratoma containing neuronal tissue was detected in 2/5 patients. Clinical outcome at the last follow up after 8–40 months (mRS median 1.0, interquartile range 0.75-4.0, n = 6) was good with mRS 0–1 (4/6) or showed improvement to a moderate degree (1/6), while one patient did not respond to treatment.

Patients with anti-LGI1 syndrome presented with symptoms of a classic limbic encephalitis including cognitive deficits and focal seizures. Two patients showed faciobrachial dystonic seizures that were treated successfully with methylprednisolone and antiepileptic therapy [28]. Routine EEG at the time of PET did not record epilepsy specific potentials. PET tumor screening was negative for neoplasia within a range of 1–12 months after clinical onset, which corresponds to the time from onset to treatment initiation. Again this variability of time points was caused by late admission of two patients to Hannover Medical School, however, 3/4 patients were at the clinical peak of the anti-LGI1 encephalitis at the time of PET with a mRS of 2.PET abnormalities of anti-LGI1 syndrome patients did not correspond to their MRI lesions that were not enhancing after gadolinium application: Multiple microangiopathic T2-hyperintensities in the subcortical white matter (patient 1) and bitemporal T2-hyperintensities (patient 2, Figure 1C-D). At the time of PET, the MRI of patient 3 had not displayed any lesions yet, while we detected a right hippocampal T2-hyperintensity 4 months later corresponding to progressive cognitive deficits (memory, executive functions) and generalized seizures, patient 4 did not show MRI lesions.

After first line treatments, two patients received a continuous oral steroid therapy, while patient 3 was treated with cyclophosphamide at mRS of 3. In most patients clinical outcome at the last follow up after 9–31 months (mRS median 0.5, interquartile range 0.25-2.5, n = 4) was good with mRS 0–1 (3/4).

### Group analyses of brain glucose metabolism

**Figure 2:**
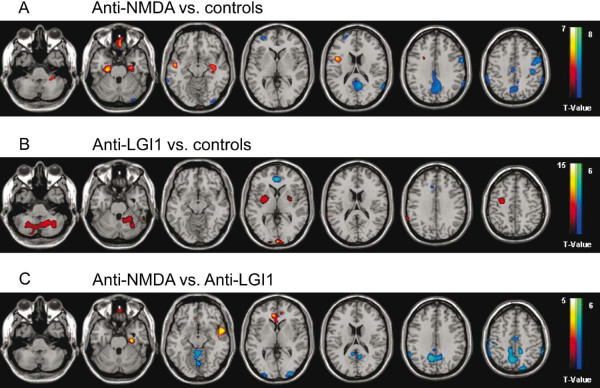
^**18**^**F-fluoro-2-deoxy-d-glucose positron emission tomography in patients with anti-N-methyl-D-aspartate receptor and anti-leucine rich glioma inactivated 1 protein encephalitis – tomographic display.** Group analysis by statistical parametrical mapping of ^18^F-fluoro-2-deoxy-d-glucose positron emission tomography shows significant (p < 0.005, two sample t-test uncorrected for multiple comparisons and an extent threshold of 30 voxels) hypermetabolism and hypometabolism in different brain regions of investigated patients with anti-N-methyl-D-aspartate receptor encephalitis (A, anti-NMDA, n = 6) and anti-leucine rich glioma inactivated 1 protein encephalitis (B, anti-LGI1, n = 4) compared to age and sex matched controls (n = 5–6). **A**, Clusters of significant voxels projected onto magnet resonance tomograms in Montreal Neurological Institute space illustrate the typical pattern of temporomesial hippocampal and parahippocampal hypermetabolism as well as widespread hypometabolism in the precuneus, pre- and postcentral, parietal and posterior cingulate cortex of anti-N-methyl-D-aspartate receptor encephalitis patients. **B**, In anti-leucine rich glioma inactivated 1 protein encephalitis we observed hypermetabolism in the cerebellum, basal ganglia, precentral and occipital areas and hypometabolism limited to the anterior cingulate / frontomesial cortex. **C**, Direct comparison of anti-N-methyl-D-aspartate and anti-leucine rich glioma inactivated 1 protein encephalitis patients revealed hypometabolism in the precuneus, parietal, occipital and cingulate cortex and hypermetabolism limited to frontotemporal regions. Significant hypermetabolism is indicated by red to yellow and hypometabolism by blue to green color-coding.

**Figure 3:**
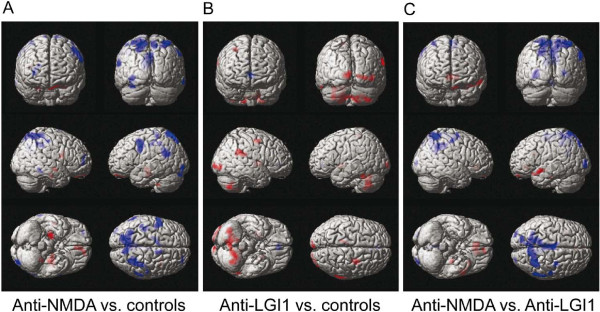
^**18**^**F-fluoro-2-deoxy-d-glucose positron emission tomography in patients with anti-N-methyl-D-aspartate receptor and anti-leucine rich glioma inactivated 1 protein encephalitis – projection onto surface display.** Group analysis by statistical parametrical mapping of ^18^F-fluoro-2-deoxy-d-glucose positron emission tomography in patients with anti-N-methyl-D-aspartate receptor (anti-NMDA, n = 6) and anti-leucine rich glioma inactivated 1 protein encephalitis (anti-LGI1, n = 4) displays significant (p < 0.005, two sample t-test uncorrected for multiple comparisons and an extent threshold of 30 voxels) hypermetabolism and hypometabolism in different brain regions compared to matched controls (n = 5–6). **A**, Clusters of significant voxel projected onto magnet resonance based images of the brain surface demonstrate regionally limited hypermetabolism in frontotemporal areas contrasting extensive hypometabolism in parietal lobes of anti-N-methyl-D-aspartate receptor encephalitis. **B**, In anti-leucine rich glioma inactivated 1 protein encephalitis hypermetabolism is predominant in the cerebellum, basal ganglia, the occipital and precentral cortex whereas hypometabolism is restricted to the anterior cingulate/frontomesial cortex. **C**, Direct comparison between anti-N-methyl-D-aspartate receptor and anti-leucine rich glioma inactivated 1 protein encephalitis patients reveals distinct patterns of extensive hypometabolism in the precuneus, the parietooccipital and posterior cingulate cortex as well as hypermetabolism in frontomesial and temporal areas. Significant hypermetabolism is indicated by red and hypometabolism by blue color coding.

In comparison to the matched reference group FDG-PET of patients with anti-NMDA receptor encephalitis (Figures 2A, 3A) displayed a regionally limited hypermetabolism of bitemporal areas including hippocampus and parahippocampus (99–439 voxels within Brodmann areas 20, 21, 22, 30, 35, 36, 48, Table 3) as well as an extensive hypometabolism in the precuneus, the pre- and postcentral, the parietal and posterior cingulate cortex (503–1821 voxels within Brodmann areas 2, 3, 4, 6, 7, 17, 23, 26, 29, 30, 40, 43, Table 4). In patients with anti-LGI1 encephalitis (Figures 2B, 3B) we observed hypermetabolism in the cerebellum, basal ganglia as well as in precentral and occipital regions (191–2286 voxels in the cerebellum, putamen, pallidum and within Brodmann areas 6, 8, 17, 18, 19, Table 4) and hypometabolism limited to the anterior cingulate/frontomesial cortex (232 voxels within Brodmann areas 10, 11, 24, 32, Table 4). Brain regions and Brodmann areas showing significant abnormalities of glucose metabolism in patients with anti-LGI1 and anti-NMDA receptor encephalitis are summarized in Table 3 and Table 4. The results of SPM analysis for each individual patient are displayed in the supplemental material (Additional files 2 and 3: Figures S1 and S2 and Additional file 4: Table S2).

Direct comparison between the above mentioned types of encephalitis likewise revealed distinct patterns with a relative hypometabolism in the precuneus, precentral, parietal, occipital and cingulate cortex (338–2616 voxels within Brodmann areas 2, 3, 4, 5, 6, 7, 10, 19, 20, 23, 26, 30, 39, 40) as well as regionally limited hypermetabolism in frontotemporal areas (40–449 voxels within Brodmann areas 10, 11, 20, 21, 22, 25, 30, 36, 38, 47, 48) of anti-NMDA receptor encephalitis patients compared to the anti-LGI1 associated syndrome (Figures 2C, 3C).

**Table 3:** ^**18**^**F-fluoro-2-deoxy-d-glucose positron emission tomography in anti-N-methyl-D-aspartate receptor encephalitis patients**

**Hypermetabolism**	**Hypometabolism**
Hippocampus, parahippocampal, temporal sup., fusiform gyrus left	Precuneus bilat., post. + mid. cingulum bilat., cuneus, calcarine left
(439 voxels in BA 20, 30, 35, 36, 48)	(1821 voxels in BA 7, 17, 23, 26, 29, 30)
Hippocampus, parahippocampal right	Parietal superior, precuneus bilat.
(264 voxels in BA 20, 30, 35, 36)	(896 voxels in BA 5, 7)
Temporal sup., mid. right	Parietal sup. bilat., precuneus bilat. parietal inf., postcentral right
(99 voxels in BA 20, 21, 22, 48)	(720 voxels in BA 2, 3, 5, 7, 40)
Frontal inf., operculum, Insula right	Pre-, postcentral, frontal mid. left
(57 voxels in BA 6, 48)	(503 voxels in BA 4, 6, 9, 43)
Gyrus rectus, frontal supra-orbital, left	Frontal mid, sup. right
(47 voxels in BA 11)	(149 voxels in BA 10, 11, 46, 47)
Cerebellum left	Occipital mid., inf. lingual, left
(47 voxels)	(145 voxels in BA 18, 19)
	Parietal supramarginal, inf. right
	(93 voxels in BA 40)
	Parietal supramarginal, temporal sup., mid. left
	(77 voxels in BA 22)
	Postcentral left
	(73 voxels in BA 1, 2 ,3 ,4)
	Frontal sup. left, suppl. mot. area bilat.
	(60 voxels in BA 6)
	Temporal inf., mid. right
	(48 voxels in BA 20, 37)

*Abbreviations:* Brodmann area (BA).

Brain regions and Brodmann areas displaying significant abnormalities of glucose metabolism in ^18^F-fluoro-2-deoxy-d-glucose positron emission tomography of patients with anti-N-methyl-D-aspartate receptor encephalitis (n = 6, p < 0.005, two sample t-test uncorrected for multiple comparisons and an extent threshold of 30 voxels) compared to age and sex matched controls (n = 5).

**Table 4:** ^**18**^**F-fluoro-2-deoxy-d-glucose positron emission tomography in patients with anti-leucine rich glioma inactivated 1 protein encephalitis**

**Hypermetabolism**	**Hypometabolism**
Cerebellum, bilat.	Cingulum ant. bilat., mid. frontal sup. med., med. orbital right
(2286 voxels)	(232 voxels in BA 10, 11, 24, 32)
Putamen, pallidum right	
(322 voxels)	
Precentral, frontal mid., sup. right	
(210 voxels in BA 6, 8)	
Putamen, pallidum left	
(191 voxels)	
Occipital inf., mid., calcarine right	
(191 voxels in BA 17, 18, 19)	
Parietal supramarginal, angular, temporal sup. right	
(155 voxels in BA 22, 40, 48)	
Calcarine, occipital mid., sup. left	
(149 voxels in BA 17)	
Paracentral lobule, precuneus left	
(56 voxels in BA 2, 4, 5)	
Temporal inf. left	
(37 voxels in BA 20, 37)	

*Abbreviations:* Brodmann area (BA).

Brain regions and Brodmann areas displaying significant abnormalities of glucose metabolism in ^18^F-fluoro-2-deoxy-d-glucose positron emission tomography of patients with anti-leucine rich glioma inactivated 1 protein encephalitis (n = 4, p < 0.005, two sample t-test uncorrected for multiple comparisons and an extent threshold of 30 voxels) in comparison to matched controls (n = 6).

In LGI1-patients the mRS at follow up showed a positive correlation with the precentral metabolism (i.e. increasing mRS values with increasing frontal metabolism left, r = 0.95, p = 0.048). Other assessments for correlations between brain metabolism and NMDA-/LGI1-antibody titers in serum/CSF or the mRS at follow up did not reveal consistent results of SPM and VOI analyses.

## Discussion

This retrospective study showed distinct brain metabolic patterns in FDG-PETs of anti-LGI1 and anti-NMDA receptor encephalitis patients. Analysis of the anti-LGI1 group revealed hypermetabolism in cerebellar, basal ganglia, occipital and precentral regions and hypometabolism limited to the anterior cingulate/frontomesial cortex, whereas in anti-NMDA receptor encephalitis we found hypermetabolism in frontotemporal areas and widespread hypometabolism in parietal lobes.

The imaging results for anti-NMDA receptor encephalitis patients are in agreement with a recent FDG-PET study demonstrating frontotemporal hypermetabolism and occipital hypometabolism [23] despite considerable methodological differences. Our anti-NMDA receptor encephalitis patients with mRS ≥ 4 at the time of PET (n = 5) had to receive propofol narcosis during the whole-body tumor scan to prevent severe artifacts by involuntary movements, while Leypoldt *et al*. managed imaging acquisition in patients showing similar initial mRS (median 4.5) without sedation or narcosis (F. Leypoldt, personal communication).

The tracer FDG is taken up by active brain neurons as if it was glucose and is then metabolized in the cells to FDG-6-phosphate emitting radioactivity that can be measured using PET. Uptake and metabolic trapping of FDG in the brain as FDG-6-phosphate is completed to 80–90% 32 minutes after the intravenous injection [29]. In an FDG-PET study with healthy volunteers receiving narcosis 25 min before FDG administration and during PET scanning, the regional glucose metabolic rate was reduced during propofol anaesthesia in all brain areas to 48–66% (p < 0.01) with highest significant reductions in the occipital lobe, the lingual gyrus, parietal lobe, temporal lobe and thalamus [30]. Although we applied FDG one hour before initiation of narcosis and PET scanning, we cannot completely rule out a minor hypometabolic propofol effect on brain metabolism particularly in temporal and parietal areas of our anti-NMDA receptor encephalitis patients. In most affected temporal regions including hippocampus and parahippocampus an underestimation of encephalitis-induced hypermetabolism due to propofol narcosis seems possible. In previous FDG-PETs without sedation or narcosis, frontotemporal hypermetabolism was more pronounced in anti-NMDA receptor encephalitis with similar mRS [23] suggesting a minor hypometabolic propofol effect in these brain areas of our patients.

VGKC-complex positive encephalitis patients without LGI1 antibodies compose a more heterogenous group than patients with LGI1 encephalitis. In a recent PET study [31], only 1/7 VGKC-positive patients also showed autoantibodies against LGI1 leading to hypometabolism in the association cortices. In the remaining six patients, two scans were rated as normal and four showed different findings mostly involving the basal ganglia [31] suggesting a heterogeneous pattern of brain glucose metabolism in the VGKC-positive subtypes of limbic encephalitis. In previous case reports, the FDG-PETs in encephalitis patients with autoantibodies against the VGKC-complex – LGI1 antibodies were not determined – indicated bilateral temporomesial hypermetabolism and/or temporal hypometabolism depending on the course of the disease [32-35].

The glycoprotein LGI1 is secreted from presynaptic terminals and associates with synaptic Kv1 VGKCs [36]. LGI1 is highly expressed in the hippocampus and the neocortex, and mutations in the LGI1 gene cause autosomal dominant lateral temporal lobe epilepsy [13,37,38]. LGI1 antibodies have been detected predominantly in limbic encephalitis, epilepsy and few patients with Morvan’s disease [5,13,36]. Furthermore, LGI1 antibodies were detected in a glutamic acid decarboxylase antibody-positive patient suffering from progressive encephalomyelitis with rigidity and myoclonus (PERM) [39].

Recent case reports demonstrated hypermetabolism in the basal ganglia as well as in the left hippocampus and amygdala in two patients 3–8 months after clinical onset of encephalitis associated with LGI1-antibodies [17,40]. Very lately Shin *et al*. demonstrated temporal and bilateral basal ganglia hypermetabolism in 7/10 anti-LGI1 encephalitis patients (3 days to 2 years between symptom onset and diagnosis), which had not been compared to a matched control group [24], suggesting that the anti-LGI1-induced brain metabolic pattern may depend on the disease course, time point of diagnosis/therapy initiation and treatment regimen.

Our anti-LGI1 patients were investigated without propofol narcosis by FDG-PET 1–12 months after clinical onset showing only minor hypermetabolism in temporal areas, whereas hypermetabolic activity was most pronounced in cerebellar, basal ganglia, occipital and precentral regions. Interestingly, the precentral hypermetabolism correlated positively with the mRS at follow up suggesting that increased metabolism in this area may represent a negative prognostic factor in LGI1 patients. The anti-LGI1 metabolic pattern contrasts the PET results of patients with anti-NMDA receptor encephalitis underlining these two etiologically different subtypes of autoimmune limbic encephalitis. In addition to the autoantibody detection as most important diagnostic feature, FDG-PET may prove to be a useful imaging tool, besides whole-body tumor search, for differentiating these subtypes of limbic encephalitis.

Two distinct brain metabolic patterns in immunologically mostly undefined (7/9) limbic encephalitis patients have been described in a recent FDG-PET study [41]. Five younger patients including the two NMDA-receptor antibody positive cases displayed a mixed metabolic pattern characterized by hypermetabolism in the temporal and orbitofrontal cortex as well as occipital hypometabolism. While this pattern in younger patients reminds of brain metabolic changes seen in limbic encephalitis induced by NMDA receptor IgG-antibodies [23], four older patients presented with subacute cognitive decline showing diffuse cortical hypometabolism closely resembling Alzheimer’s disease or dementia with Lewy Bodies [41]. This neurodegenerative-like hypometabolism is neither likely to reflect the cognitive decline induced by NMDA receptor IgA-antibodies leading to occipital hypermetabolism [42] nor the anti-LGI1 syndrome where Shin *et al*. and we found predominantly hypermetabolic changes.

## Conclusions

In conclusion, our retrospective FDG-PET study provides novel evidence for distinct brain metabolic patterns in patients with anti-LGI1 and anti-NMDA receptor encephalitis. In anti-NMDA receptor encephalitis the regionally limited hypermetabolism in frontotemporal areas contrasted extensive hypometabolism in parietal lobes, whereas the anti-LGI1 syndrome is characterized by hypermetabolism in cerebellar, basal ganglia, occipital and precentral areas and minor frontomesial hypometabolism.

### Ethical standard

We obtained an approval from the local Ethics Committee of Hannover Medical School (No. 1625–2012) and patients or their carers gave their written informed consent.

## Abbreviations

ADAM22: Disintegrin and metalloproteinase domain-containing protein 22; AMPA: α-amino-3-hydroxy-5-methyl-4-isoxazolepropionic acid; BA: Brodmann area; CSF: Cerebrospinal fluid; CT: Computed tomography; EEG: Electroencephalography; FDG-PET: ^18^F-fluoro-2-deoxy-d-glucose positron emission tomography; FLAIR: Fluid attenuated inversion recovery; FWHM: Full width at half maximum; IgG: Immunoglobulin G; LGI1: Leucine rich glioma inactivated 1 protein; MRI: Magnet resonance imaging; mRS: Modified Rankin Scale; NMDA: N-methyl-D-aspartate; NR: NMDA receptor subunit; OSEM: Ordered subset expectation maximization; SPM: Statistical parametric mapping; VOI: Volume of interest; VGKC: Voltage-gated potassium channels.

## Competing interests

The authors declare that they have no competing interest.

## Authors’ contributions

FWe, FWi, PR, CH, GB, EN: study design and data interpretation. FWe, FWi, PR, SBT, CH, RRR, GB, EN: data analysis. FWe, SBT, ALB, CT, MS, EV, CS, FL, JA, AL: patient selection and treatment. FWe, FWi, PR, RD, LG, FMB, GB, EN: manuscript drafting. All authors read and approved the final manuscript.

## Pre-publication history

The pre-publication history for this paper can be accessed here:

http://www.biomedcentral.com/1471-2377/14/136/prepub

## Supplementary Material

Additional file 1: Table S1 Volume of interest analyses of ^18^F-fluoro-2-deoxy-d-glucose positron emission tomography in patients with anti-N-methyl-D-aspartate receptor and anti-leucine rich glioma inactivated 1 protein encephalitis. Click here for file

Additional file 2: Figure S1 ^18^F-fluoro-2-deoxy-d-glucose positron emission tomography images of individual patients with anti-N-methyl-D-aspartate receptor encephalitis. Click here for file

Additional file 3: Figure S2 ^18^F-fluoro-2-deoxy-d-glucose positron emission tomography images of individual patients with anti-leucine rich glioma inactivated 1 protein encephalitis. Click here for file

Additional file 4: Table S2 ^18^F-fluoro-2-deoxy-d-glucose positron emission tomography data of individual patients with anti-N-methyl-D-aspartate receptor and anti-leucine rich glioma inactivated 1 protein encephalitis. Click here for file
