# Supplementary material for: Anti-leucine rich glioma inactivated 1 protein and anti-N-methyl-D-aspartate receptor encephalitis show distinct patterns of brain glucose metabolism in 18F-fluoro-2-deoxy-d-glucose positron emission tomography
Source: BMC Neurol. 2014 Jun 20;14:136. doi: 10.1186/1471-2377-14-136 (PMC4076767; doi:10.1186/1471-2377-14-136)
Supplement: Additional file 1: Table S1 — Volume of interest analyses of 18F-fluoro-2-deoxy-d-glucose positron emission tomography in patients with anti-N-methyl-D-aspartate receptor and anti-leucine rich glioma inactivated 1 protein encephalitis. [file 1471-2377-14-136-S1.pdf]

Region	Anti-NMDA PET vs. controls<br>p	Anti- LGI1 PET vs. controls<br>p	Anti-NMDA PET vs. Anti-LGI1 PET<br>p
Central right			0,0386
Central left	0,0375		
Thalamus right			
Thalamus left			
Temporal right			
Temporal left			
Striat./Pall. right			
Striat./Pall. Left			
Parietal right	0,0166		0,0026
Parietal left	0,0154		0,0489
Occipital right			
Occipital left	0,0493		
mes. Temp. right	0,0173		
mes. Temp. Left	0,0160		
Frontal right			
Frontal left		0,0460	
Cingulum right			
Cingulum left	0,0427	0,0248	
Cerebellum right			
Cerebellum left		0,0398	

Significant Hypometabolism in patients (blue)

Significant Hypermetabolism in patients (red)

(Blue cells: 0,0386; 0,0375; 0,0166; 0,0026; 0,0154; 0,0489; 0,0493; 0,0460; 0,0427; 0,0248. Red cells: 0,0173; 0,0160; 0,0398.)
