# Supplementary figures and images for: Anti-leucine rich glioma inactivated 1 protein and anti-N-methyl-D-aspartate receptor encephalitis show distinct patterns of brain glucose metabolism in 18F-fluoro-2-deoxy-d-glucose positron emission tomography
Source: BMC Neurol. 2014 Jun 20;14:136. doi: 10.1186/1471-2377-14-136 (PMC4076767; doi:10.1186/1471-2377-14-136)

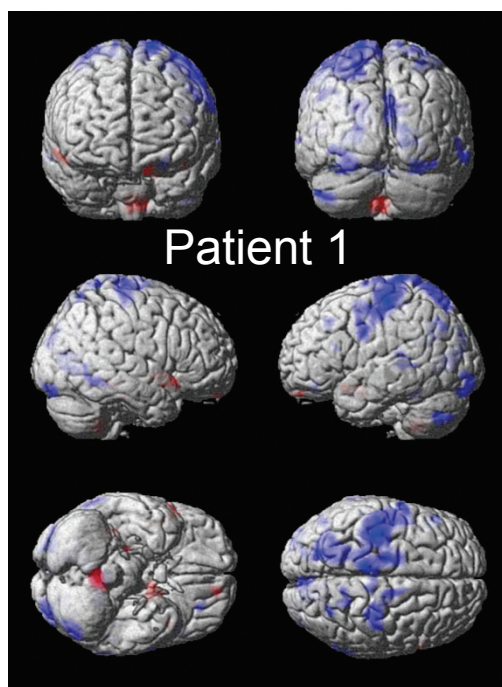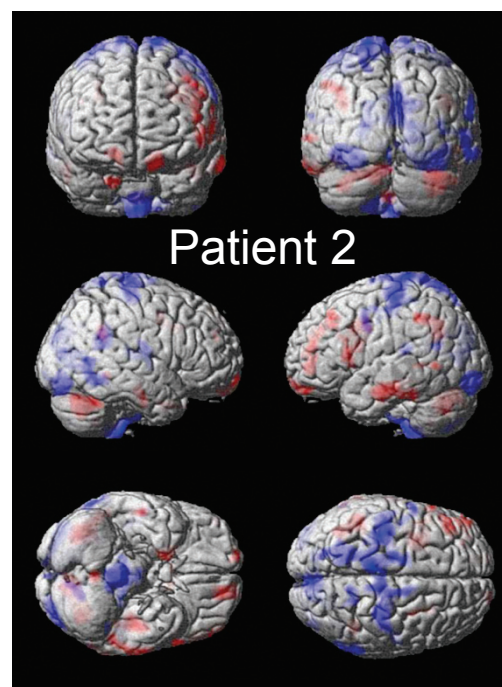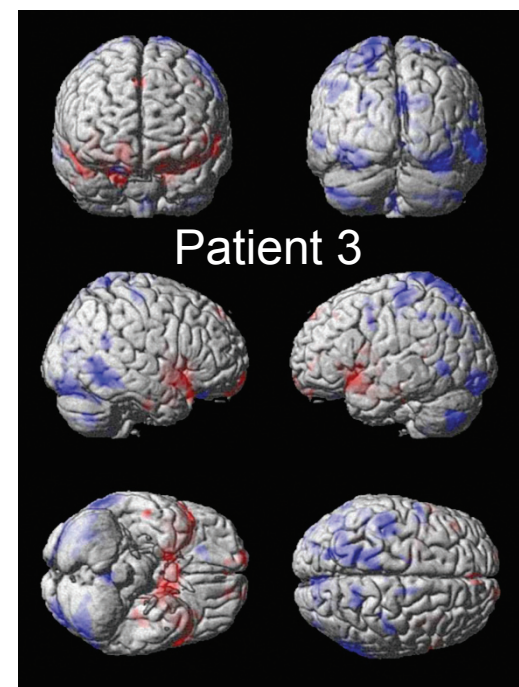

**Anti-NMDA**

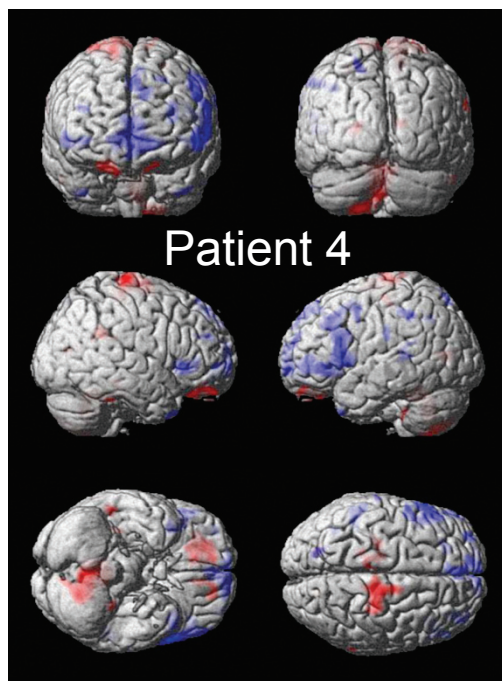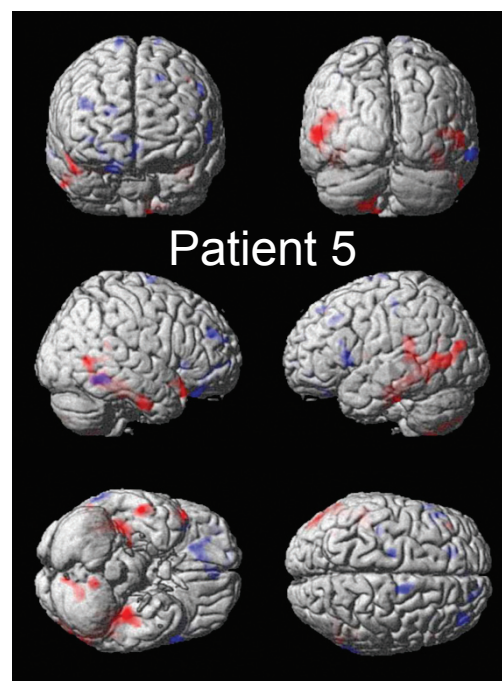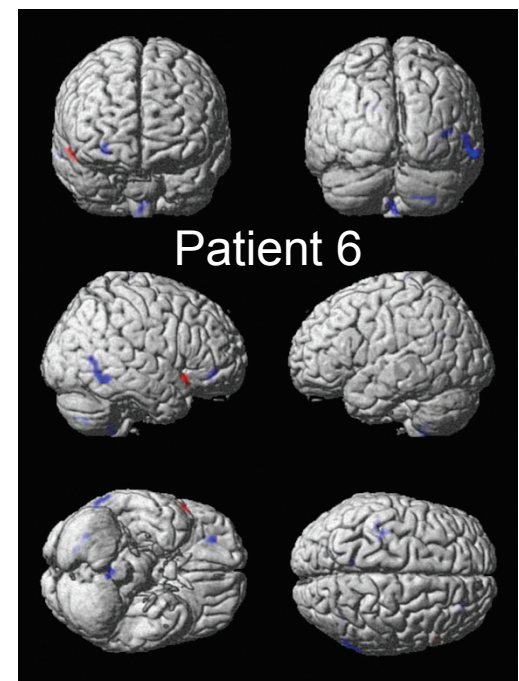

Supplement: Additional file 2: Figure S1 — 18F-fluoro-2-deoxy-d-glucose positron emission tomography images of individual patients with anti-N-methyl-D-aspartate receptor encephalitis. [file 1471-2377-14-136-S2.pdf]

Patient 1

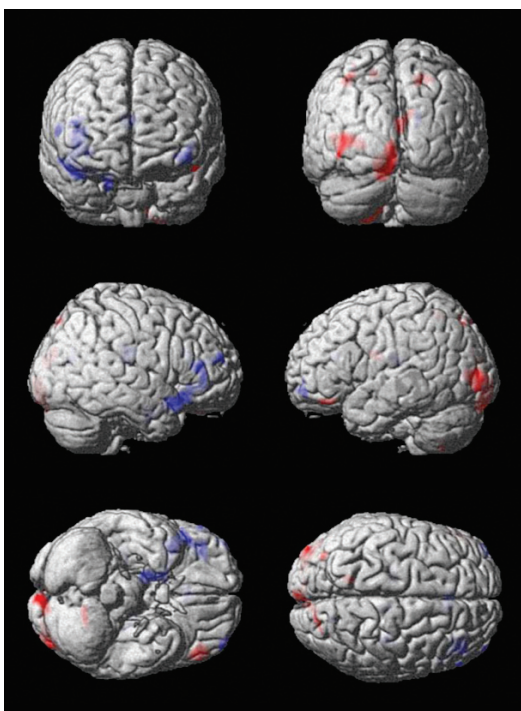

**Anti-  
LGI1**

Patient 2

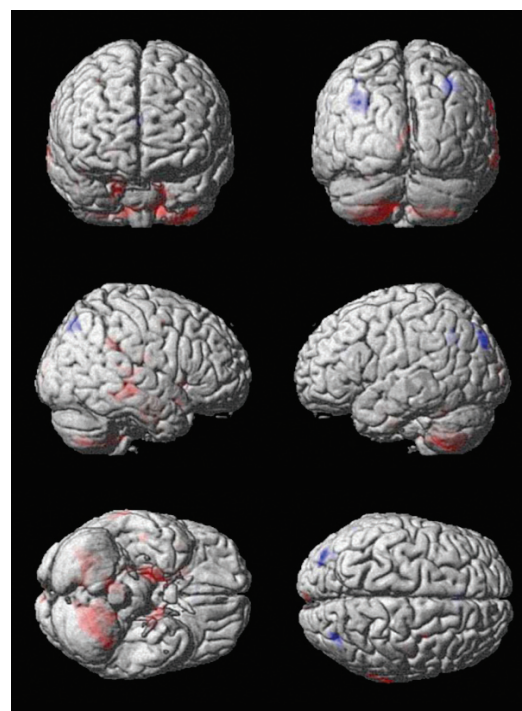

Patient 3

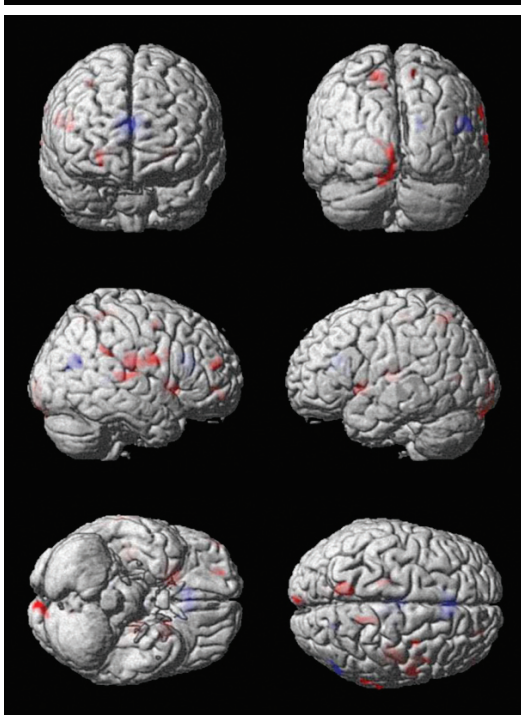

Patient 4

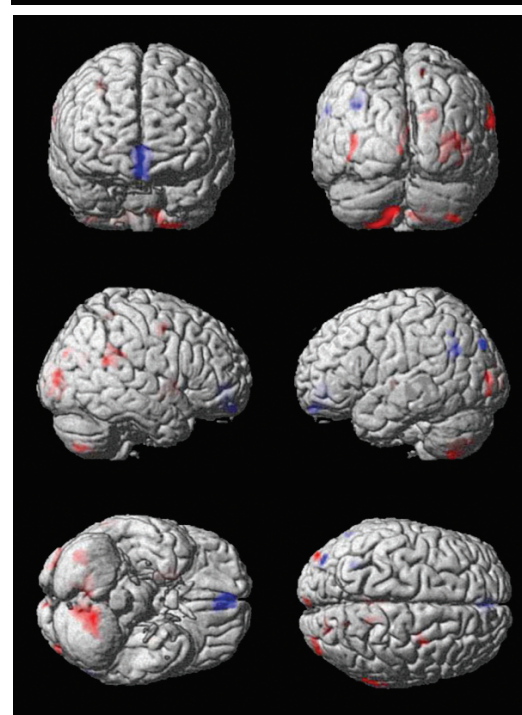

Supplement: Additional file 3: Figure S2 — 18F-fluoro-2-deoxy-d-glucose positron emission tomography images of individual patients with anti-leucine rich glioma inactivated 1 protein encephalitis. [file 1471-2377-14-136-S3.pdf]
