# Supplementary material for: Anti-leucine rich glioma inactivated 1 protein and anti-N-methyl-D-aspartate receptor encephalitis show distinct patterns of brain glucose metabolism in 18F-fluoro-2-deoxy-d-glucose positron emission tomography
Source: BMC Neurol. 2014 Jun 20;14:136. doi: 10.1186/1471-2377-14-136 (PMC4076767; doi:10.1186/1471-2377-14-136)
Supplement: Additional file 4: Table S2 — 18F-fluoro-2-deoxy-d-glucose positron emission tomography data of individual patients with anti-N-methyl-D-aspartate receptor and anti-leucine rich glioma inactivated 1 protein encephalitis. [file 1471-2377-14-136-S4.pdf]

Group	Hyper-/Hypometabolism	Localisation	Patient-No.	Frequency
**Anti-NMDA**	Hypermetabolism	Hippocampal	1, 2, 3, 5	67%
		Parahippocampal	1, 2, 3, 4, 5	83%
	Hypometabolism	Precuneus	1, 2, 3, 6	67%
		Parietal	1, 2, 3, 4, 5	83%
		Post. Cingulum	6	17%
		Central	1, 2, 3, 5	67%
**Anti-LGI1**	Hypermetabolism	Cerebellum	1, 2, 4	75%
		Putamen/Pallidum	2, 3, 4	75%
		Precentral	2, 3, 4	75%
		Occipital	1, 4	50%
	Hypometabolism	Ant. Cingulum / Front. med.	1, 2, 3, 4	100%
